# Genetic Toxicology and Safety Pharmacological Evaluation of Forsythin

**DOI:** 10.1155/2021/6610793

**Published:** 2021-06-18

**Authors:** Zhong Han, Jianmin Guo, Feibiao Meng, Haifeng Liao, Yinghua Deng, Yuankeng Huang, Xialing Lei, Chun Liang, Richou Han, Wei Yang

**Affiliations:** ^1^Guangdong Key Laboratory of Animal Conservation and Resource Utilization, Guangdong Public Laboratory of Wild Animal Conservation and Utilization, Institute of Zoology, Guangdong Academy of Sciences, Guangzhou, Guangdong, China; ^2^Guangdong Lewwin Pharmaceutical Research Institute Co., Ltd., Guangdong Provincial Key Laboratory of Drug Non-Clinical Evaluation and Research, Guangdong Engineering Research Center for Innovative Drug Evaluation and Research, Guangzhou, Guangdong, China; ^3^Division of Life Science, Center for Cancer Research and State Key Lab for Molecular Neuroscience, Hong Kong University of Science and Technology, Hong Kong, China; ^4^EnKang Pharmaceuticals (Guangzhou) Limited, Guangzhou, China

## Abstract

**Introduction:**

Forsythin is the main ingredient of *Forsythia suspensa* and is widely used in treatment of fever, viral cold, gonorrhea, and ulcers clinically. This study aimed to evaluate the potential genetic toxicity of forsythin and its safety for human use.

**Methods:**

Based on the Good Laboratory Practice regulations and test guidelines, the genetic toxicity of forsythin was assessed by the Ames test, chromosome aberration (CA) test, and bone marrow micronucleus (MN) test in vivo. In the Ames test, five strains of *Salmonella typhimurium* were exposed to different concentrations of forsythin in the presence or absence of the S9 mixture, and then, the number of His + revertant colonies was counted. In the CA test, Chinese hamster lung (CHL) fibroblast cells were treated with different concentrations of forsythin, mitomycin C, or cyclophosphamide in the presence or absence of the S9 mixture, and the chromosomal aberrations were determined. In the MN test, bone marrow was isolated from the mice with different treatments, and the ratios of polychromatic erythrocytes (PCE) and erythrocytes (PCE/(PCE + NCE)) were measured. Finally, beagle dogs were divided into four groups (negative control, low dose, medium dose, and high dose groups), and then, a telemetry system was used to evaluate the safe use of forsythin.

**Results:**

Ames test results showed that the number of colonies in all test strains with different treatments showed no significantly dose-dependent increase in the presence or absence of the S9 mixture (*p* > 0.05). In the CA test, the number of cells with aberrations in the CHL fibroblast cells treated with low, medium, and high doses of forsythin for 24/48 h in the absence of the S9 mixture was, respectively, 5.0/2.5, 4.5/1.5, and 5.0/5.0, and in the presence of the S9 mixture, the number was, respectively, 5.0, 5.0, and 4.5. These results showed that there was no significant difference compared to the negative control group either in the presence (2.0) or in the absence (4.0/2.5 for 24/48 h) of the S9 mixture (*p* > 0.05). The MN test showed that the values of PCE/(PCE + NCE) in the negative, positive controls, and forsythin treatment groups were all more than 20%, which indicated that forsythin had no cytotoxicity. Additionally, no significant toxicological effects of forsythin on blood pressure, respiration, temperature, electrocardiogram, and other physiological indicators in the conscious beagle dogs of different groups were observed by the telemetry method.

**Conclusion:**

Our findings showed that forsythin has low probability of genetic toxicity and no significant toxicological effects, which implied that forsythin is suitable for further development and potential application.

## 1. Introduction

The clinical application of traditional Chinese medicine (TCM) has a long history in China. Due to the therapeutic effects and unique ability of TCM in treating health problems that Western medicines cannot solve, TCM has been attracting more and more attention all over the world [1–3]. It has been reported that about 4 billion people in the world are using TCM for medical treatment, accounting for 80% of the world's population [4]. Historical understanding of herbs has led people to believe that natural therapies are safer than other medicines. With the widespread of the TCM, it is essential to further investigate the efficacy and safety of TCM.

*Forsythia suspensa* (Thunb.) Vahl., belonging to the genus forsythia, the family Oleaceae, is widely distributed in China, Korea, Japan, and some European countries [5]. *Forsythia suspensa*, served as a TCM, is widely used in treatment of fever, viral cold, gonorrhea, and ulcers clinically [6]. Forsythin, the main ingredient in *Forsythia suspensa*, is an important index to determine the quality of *Forsythia suspensa*. Previous studies have shown that the contents of effective components, including forsythin, forsythiaside, and oleanolic acid, in the leaves of *Forsythia suspensa* were higher than that in its fruits [7, 8]. From the perspective of pharmacoeconomics, the development and utilization of forsythin extracted from the leaves of *Forsythia suspensa* will greatly improve the medicinal and economic value of this medicinal material resource. In addition, forsythin has been reported to have many pharmacological properties, including antibacterial, antioxidative, antiviral, hypolipidemic, hepatoprotective, anti-inflammatory, antioxidative stress, and antiapoptotic properties [6, 9, 10]. Yang et al. [11] showed that forsythin could reduce neutrophil infiltration and tissue necrosis of the zebrafish induced by lipopolysaccharide (LPS) through regulating the expression of inflammatory cytokines (IL-6, IL-1*β*, and NF-kB). Another study has indicated that forsythin combined with autophagy blockers could inhibit the proliferation of human laryngeal carcinoma epithelial cells (HEp-2 cells) and induced their apoptosis, which may be a novel strategy in treatment of laryngeal squamous cell carcinoma [12]. However, the safety of forsythin has not been fully elucidated.

The Ames test evaluates mutation of a test substance by detecting base substitution or frame shift mutations in a target gene [13]. Chinese hamster lung (CHL) fibroblast cells are one of the commonly used cell lines for studying chromosome abnormalities [14]. Micronucleus is the genetic material that remains outside of the cell nucleus at mitosis anaphase due to intracellular chromosomal disruption or spindle fragmentation. Additionally, it is recommended to use a telemetry system in conscious animals, which can eliminate the interference of anesthesia and animal activity restriction and improve the sensitivity of the test in safety pharmacology parameters [15]. Therefore, in this study, the genetic toxicity and safe use of forsythin were explored by the Ames test, chromosomal aberrations (CA) test, bone marrow micronucleus (MN) test, and telemetry method, based on the China Food and Drug Administration (CFDA) and Organization for Economic Cooperation and Development (OECD) Good Laboratory Practice (GLP) regulations and test guidelines.

## 2. Materials and Methods

### 2.1. Test Substance and Experimental Animals

**Figure 1:**
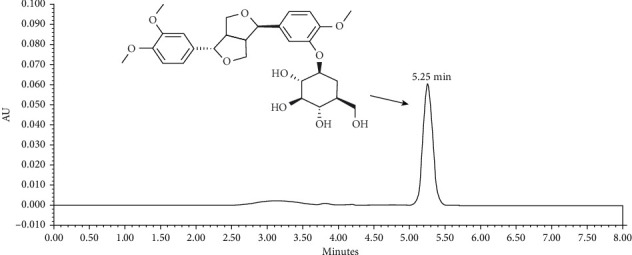
The diagram of forsythin determined by high-performance liquid chromatography-UV (HPLC-UV).

Forsythin (batch no. 20160401) was provided by Dalian Fusheng Pharmaceutical Co., Ltd. (Liaoning, China), and its purity was determined by high performance liquid chromatography-UV (HPLC-UV) as previously described [16]. Briefly, a Symmetry Shield C18 column (4.6 × 250 mm, 5 *μ*m, Ireland) and the analysis system (Waters Corporation, MA, USA) were used. The mobile phase was prepared by water and acetonitrile (V/V: 68/32), and the signal of peak was detected at the wavelength of 277 nm. The HPLC diagram of forsythin is shown in Figure 1, and the purity of forsythin was 91.61%.

Institute of Cancer Research (ICR) mice were purchased from Hunan SJA Laboratory Animal Co., Ltd. (Hunan, China), and beagle dogs were obtained from Fuzhou Zhenhe Experimental Animal Technology Development Co., Ltd. (Fujian, China). All animals were quarantined and acclimatized for a week before experiments. During the mice experiments, polypropylene was used as the feeding cage material, and the size of the cage was 320 mm × 202 mm × 135 mm. The feeding density of mice was 3–6 in each cage. Corncob bedding or poplar bedding was adopted, the ambient temperature of the animal room was 20–26°C, the relative humidity was 40–70%, and the minimum ventilation times was 15 times/h, with a 12/12 h for light/dark cycle. For beagle dog experiments, the beagle dog cages were made of stainless steel with a size of 100 cm × 120 cm × 90 cm, and each dog was kept in a single cage during the experiment. The ambient temperature of the animal room was 16–26°C, the relative humidity was 40–70%, and the ventilation times was not less than 8 times/h, with 12 h for lighting and 12 h for darkness. All the animals including ICR mice and beagle dogs have free access to food and water during the experiments. The experimental procedures were approved by the Institutional Animal Care and Use Committee of Guangdong Lewwin Pharmaceutical Research Institute Co., Ltd. (IA-SE2016014-02 and IA-SE2016015-01). The Lewwin site is fully accredited by the Association for Assessment and Accreditation of Laboratory Animal Care (AAALAC).

### 2.2. Cell Culture

CHL fibroblast cells were provided by the Chinese Academy of Military Medical Sciences (Beijing National Center for Drug Safety Evaluation and Research). The cells were cultured in minimum essential medium (GIBCO, Carlsbad, CA, USA) supplemented with 10% fetal bovine serum (FBS, GIBCO) and incubated in a humidified atmosphere containing 5% CO_2_ at 37°C.

### 2.3. Ames Test

The Ames test (bacterial reverse mutation assay) was carried out to examine the potential mutagenicity of forsythin, based on OECD GLP guideline 471 (OECD, 1997a, Test No.471) [17]. Considering the precipitate in the solution may interfere with the scoring, the highest amount of the test substance (the insolubility of forsythin) should be confirmed. The preliminary experiment found that 2500 *μ*g/plate was the highest dose of the final mixture without visible precipitation. Therefore, in the formal experiments of the Ames test, 2500 *μ*g/plate forsythin was used as the highest dose. After that, the methods of the Ames test were described previously, and the plate incorporation was used for the Ames test [18, 19]. Briefly, *Salmonella typhimurium* (ST) strains TA97, TA98, TA100, TA102, and TA1535 were obtained from the AMES Laboratory, National Center for Drug Safety Evaluation and Research, Beijing, China, and the requirements of these strains for histidine or tryptophan as well as their sensitivity to ampicillin, crystal violet, and ultraviolet radiation were checked. The genetic characteristics of the five strains were verified, and the strains were used for the mutagenesis tests. Afterwards, in the presence or absence of an S9 mixture (10%, V/V; CHI Scientific (Jiangsu, China), batch number: 16FS004), the ST strains were exposed to different concentrations of forsythin (156–2500 *μ*g/plate) at 37°C for 48 h, and then, the number of colonies was counted. Dimethylsulfoxide (DMSO) was used as the negative control. Additionally, sodium azide and Dexon (Sigma-Aldrich) in the absence of a metabolic activation system, and 2-nitrofluorene, cyclophosphamide, and 1, 8-dioxyanthraquinone (Sigma-Aldrich) in the presence of a metabolic activation system were utilized for positive controls. Compared with the negative control, a 2-fold or 3-fold increase in the number of revertant colonies in a dose-dependent manner is considered a positive result [20]. Ames test results were expressed as mean ± standard deviation and analyzed using one-way analysis of variance (ANOVA). Differences were considered statistically significant when *p* < 0.05.

### 2.4. Chromosomal Aberrations (CA) Test

The CA test were performed in accordance with CFDA guidance and OECD GLP guideline 473 (OECD, 1997b, Test No. 473) [21], and the methods of the CA test were described as previously [22] with some minor modifications. Briefly, under the condition with S9 mixture, the CHL fibroblast cells (a density of 1 × 10^4^ cells/well) were treated with different concentrations of forsythin (500, 250, and 125 *μ*g/mL), mitomycin C (0.25 *μ*g/mL), or cyclophosphamide (20 *μ*g/mL) for 6 h, and then, after washing, the cells were cultured in fresh medium for another 18 h. However, under the condition without S9 mixture, the CHL fibroblast cells (a density of 1 × 10^4^ cells/well) were treated with different concentrations of forsythin, mitomycin C, or cyclophosphamide for 24 h or 48 h. After that, 0.2 *μ*g/mL colcemid (GIBCO, Carlsbad, CA, USA) was added. After incubated for 2 h, the cells were fixed with acetic acid and methanol (1 : 3) and then stained with 4% Giemsa solution. Two hundred metaphases per dose were observed under a 1000x oil lens (DM3000, Leica, Germany), and the frequency of cells with aberrations was calculated. The frequency less than 5% was considered to be negative; for positive, the frequency was above 10% [23].

### 2.5. Bone Marrow Micronucleus (MN) Test In Vivo

MN assay was conducted as described by Muangphra and Gooneratne [24] and Yun et al. [25] and in accordance with the OECD GLP guideline 474 (OECD, 2014, Test No. 474) [26]. Bone marrow was prepared by the method of Schmind [27] with some minor modifications. First, the preliminary study showed that oral administration of forsythin did not lead to any toxic effect at 18000 mg/kg, and 18000 mg/kg was chosen as the maximum dose. Thereafter, the high dose of forsythin (18000 mg/kg) was used to administrate to the mice once, and the marrow was taken at 24, 48, and 72 h after dosing. The results showed that there was no significant difference in MNPCEs frequency among the samples at 24 h, 48 h, and 72 h, which indicated that the bone marrow would be taken from the mice at 24 h. Meanwhile, the pharmacokinetic results of forsythin showed that forsythin was rapidly absorbed in rats after administration, and the average plasma elimination half-life was 1.5 h. After that, a total of 50 male ICR mice at 4 weeks of age were randomly divided into five groups (*n* = 10): negative control, positive control, low dose, medium dose, and high dose. The mice in the low dose, medium dose, and high dose groups were orally gavaged once with 4500, 9000, and 18000 mg/kg forsythin (equivalent to 231, 462, and 923 times of the intended human dosage). The mice in the positive control group were given intragastric administration intraperitoneally with 80 mg/kg cyclophosphamide. The mice in the negative control were injected with equal 0.5% carboxymethylcellulose sodium (CMC-Na). After treated for 24 h, all mice were euthanized by cervical dislocation, and their femora were taken. The bone marrow cells were isolated from marrow and spread on glass slides to air-dry. Afterwards, the slides were fixed with methanol and then stained with a 5% Giemsa solution. Finally, a 1000x oil lens was used to count 2000 polychromatic erythrocytes (PCEs) per animal and 20000 PCEs per group to determine the number of micronucleated polychromatic erythrocytes (MNPCEs), and then, 200 cells per animal and 2000 cells per group were counted to calculate the values of PCE/(PCE + NCE) for assessing the cytotoxicity [28].

### 2.6. The Safety Pharmacologic Evaluation of Forsythin in Beagle Dogs

The safety pharmacologic evaluation of forsythin in beagle dogs was conducted according to the technical guidelines for the study of CDFA GLP (2014) [29] and ICH guideline S7A [30]. Before the experiment, six beagle dogs (half male and female) were randomly assigned to six telemetry signal transmitters. This experiment was set up in four groups: negative control (0.5% CMC-Na) and different doses of forsythin (24, 72, or 216 mg/kg, equivalent to 6, 18, and 56 times of the intended human dosage). Six animals were randomly selected and administered in a 4 × 6 Latin square sequence with a cleaning interval of not less than 4 days [31].

Before the experiment proper starts, all the dogs were dressed electrocardiograph (ECG) electrodes and tail-cuffs, which were used for the adaptive measurement of ECG and blood pressure signals, at least 2 hours per day, until the signal parameters were stabilized. During the experiment, the animals were dressed in vests, and the names and numbers of animals were set and recorded in the corresponding input positions of the software. The animals were weighed before each administration to calculate the doses. After wearing the telemetry equipment, the breath of each animal was corrected. Half an hour before drug administration, the basic physiological parameters of all animals were recorded, and then, the animals were administrated with drugs. The physiological data for the II lead ECG (heart rate and QTcF), respiration (respiratory rate and tidal volume), body temperature, and NIBP (mean arterial pressure: MP) were continuously recorded for 12 h after drug administration. The physiological data at 0 min, 5 min, 15 min, 30 min, 45 min, 1 h, 2 h, 4 h, 6 h, 8 h, and 12 h after drug administration were selected for the comprehensive analysis. The noninvasive telemetry system (ECG Auto ver. 3.3.3.8; EMKA Technologies, Paris, France) consisted of a data acquisition, and analysis software package was used in this study.

### 2.7. Statistical Analysis

All the data were reported as mean ± standard deviation (SD). One-way analysis of variance (ANOVA) was used for statistical analyses, and multiple comparisons were made by Dunnett's test using SPSS software version 19 (SPSS Inc., Chicago, IL, USA). The chi-square test was used to compare the incidences of chromosomal aberrations. The significant levels were set at *p* < 0.05.

## 3. Results

### 3.1. Ames Test Analysis

The results of the Ames test for forsythin are given in Table 1. It was clear that the number of His^+^ revertant colonies in the positive control strains was significantly higher than that in the negative control (over 2 times) in the presence or absence of a metabolic activation system (S9 mixture). This result suggested that the test conditions were stable and reliable. After treated with different concentrations of forsythin, the number of His^+^ revertant colonies in all test strains showed no significantly dose-dependent increase in the presence or absence of the S9 mixture (*p* > 0.05), and the number of His^+^ revertant colonies in the negative and positive controls was within the range of the historical control data. These results indicated that forsythin could have no mutagenic effects in the test strains.

### 3.2. CA Analysis

Compared with the negative control group, the incidence of chromosome structural aberrations (including breakages, fragments, and exchanges) in the positive control (mitomycin C and cyclophosphamide) groups was significantly increased either in the presence or in the absence of the S9 mixture after cultured for 24 or 48 h (*p* < 0.05, Table 2). After the CHL fibroblast cells were treated with forsythin at 125, 250, and 500 *μ*g/ml for 24/48 h in the absence of a S9 mixture, the cell number of cells with aberrations was 5.0/2.5, 4.5/1.5, and 5.0/5.0, respectively, which indicated that the incidence of chromosomal aberrations of forsythin had no significant difference compared to the negative control group in the absence of the S9 mixture (*p* > 0.05). Besides, after treated with forsythin at 125, 250, and 500 *μ*g/ml for 6–18 h in the presence of the S9 mixture, the cell number of cells with aberrations was, respectively, 5.0, 5.0, and 4.5, which showed no significant difference compared with the negative control group. All these results implied that either in the presence and absence of the S9 mixture, forsythin could not induce chromosome structural aberrations in the CHL fibroblast cells.

### 3.3. MN Test Analysis

In the preliminary study, no mortality and no signs of systemic toxicity were observed in the mice after oral administration of different doses of forsythin, and the sampling time (24 h) of the bone marrow cells was selected based on the preliminary experiment (data not shown). The ratios of PCE and all erythrocytes in the negative, positive controls, 4500 mg/kg, 9000 mg/kg, and 18000 mg/kg forsythin treatment groups were 44.3 ± 10.7%, 43.2 ± 7.6%, 55.4 ± 8.9%, 42.6 ± 10.1%, and 45.1 ± 3.9%, respectively. These results showed the ratios were all more than 20% (Table 3), which indicated that forsythin may have no cytotoxicity. Compared with the negative control (0.6 ± 0.4%), the rate of MNPCE in the positive control (80 mg/kg cyclophosphamide, 13.0 ± 4.1%) was significantly increased (*p* < 0.05, Table 3). Additionally, the rates of MNPCE in 4500 mg/kg, 9000 mg/kg, and 18000 mg/kg forsythin treatment groups were 1.0 ± 0.6%, 1.2 ± 0.8%, and 1.6 ± 0.8%, respectively, as well as were higher than that in the negative control group. However, their MNPCE rates were all less than 2%, which were within the normal range of the background data. Therefore, the results of the MN test for forsythin were negative.

### 3.4. The Safety Pharmacologic Evaluation of Forsythin by Telemetry in Conscious Beagle Dogs

**Figure 2:**
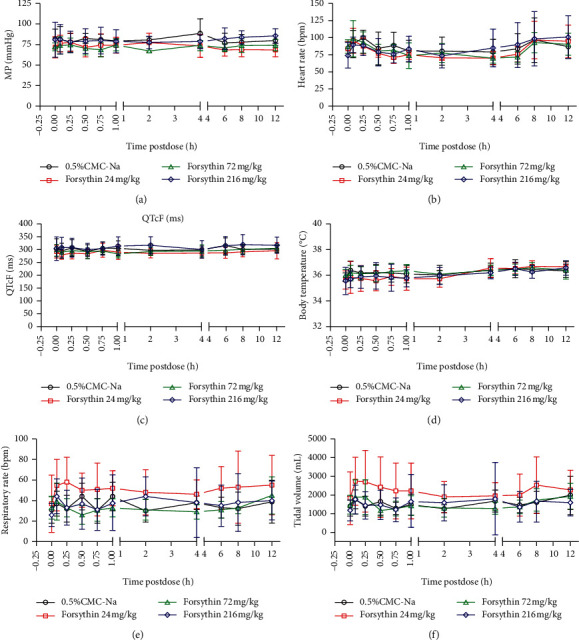
The effects of forsythin (0, 24, 72, and 216 mg/kg) on MP (a), heart rate (b), QTcF (c), body temperature (d), respiratory rate (e), and tidal volume (f) in conscious beagle dogs. All assays were conducted in compliance with Good Laboratory Practice (GLP) regulations. Data are expressed as mean ± standard deviation (SD). *n* = 6.

The effects of forsythin on the MP, heart rate, QTcF, body temperature, respiration rate, and tidal volume of conscious beagle dogs were analyzed. Before treatment, the indexes of MP, heart rate, and QTcF in beagle dogs of each group were similar, and no abnormalities were found (Figures 2(a)–2(c)). After administrated with different doses of forsythin, the MP and QTcF were shortened or prolonged, accompanied by the increased or decreased heart rate (Figures 2(a)–2(c)). The beagle dogs in the negative control also showed the above characteristics. It was inferred that these changes were of nonsignificance in term of toxicity.

In addition, the body temperature of beagle dogs was further monitored. The body temperature of beagle dogs was increased at some time points (such as 0.25 h with 72,216 mg/kg forsythin) after treated with forsythin, which had difference with that before administration (Figure 2(d)). However, there was no significant difference in the body temperature index among groups. It was concluded that the slight increase in the body temperature after drug administration was a normal physiological phenomenon.

In addition, compared with the negative control, the respiratory rate and tidal volume of beagle dogs were significantly increased or decreased at some individual time points after forsythin treatment (Figures 2(e) and 2(f)). However, there were no significant differences in the respiratory rate and tidal volume of beagle dogs before and after administrations.

## 4. Discussion

Recently, natural products have been paid more and more attentions because of their wide sources, pharmacological activities, low costs, and minimal side effects [32]. Most of the research studies have focused on the analysis of the species of natural products and their active components [33]. *Forsythia suspensa*, a natural product, has been reported to have heat-clearing and detoxifying effects and is used as a single herb and in compound prescriptions in Asia [34]. Forsythin, a representative ingredient of *Forsythia suspensa*, possesses many biological effects [5, 10, 35], including anti-inflammatory, antibacterial, antioxidant, antiviral, and neuroprotective effects. Ma et al. [36] showed that forsythin could inhibit the proliferation of COVID-19 and HCoV-229E in vitro and can be applied for controlling the coronavirus disease 2019. Additionally, the safety of *Forsythia suspensa* fruits has been evaluated. Yin et al. [37] analyzed the mutagenic potential of 102 raw drugs and found that the extraction of *Forsythia suspensa* fruits was negative in the Ames test and was positive in the CA and MN test. Another study indicated that the aqueous extract of *Forsythia viridissima* fruits showed no genotoxicity via the Ames test (5000 *μ*g/plate), MN test (5000 mg/kg), and CA test (1100–2500 *μ*g/mL) [38]. Our study was the first to investigate the genetic toxicology and safety pharmacology of forsythin. The negative results of the Ames test, CA test, and MN test in vivo showed that forsythin had no genetic toxicity. Additionally, no significant differences in MP, heart rate, QTcF, body temperature, respiratory rate, and tidal volume of conscious beagle dogs were found among control and different doses of forsythin groups, which indicated that forsythin had no toxicity and was safe. These results will reveal that forsythin may be suitable for further development and potential application. Genetic toxicology is an important part of nonclinical safety evaluation of drugs and is an important link for drug candidates to move from the discovery stage to the clinical stage and the market. In our study, the Ames test, CA test, and MN test were utilized to assess the potential genotoxicity of forsythin. Our results of the Ames test showed that in the presence or absence of the S9 mixture, the number of His + revertant colonies in all test strains after forsythin had no significant difference with the negative and positive controls. These indicated that forsythin has no mutagenic effects in the test strains. Chromosomes, consisted of DNA and proteins, are the carrier of genetic information and have unique morphologies and structures [39]. They are prone to be damaged by external mutagens, potentially causing morphological and structural distortions. The results of the CA test in our study showed that there was no significant difference in the incidence of chromosomal aberrations between the negative control and forsythin treatment groups (*p* > 0.05), which implied that forsythin could not induce chromosome aberration of cells. In addition, the genotoxic effects of test substances based on chromosome breakage and/or damage can be determined through altering the number of polychromatic erythrocytes with micronucleus in the bone marrow of rodents [40]. In this experiment, the maximum tolerated dose was 18,000 mg/kg, equivalent to 923 times of the human clinical dosage. MN test results displayed that the MNPCE rates in the forsythin treatment groups were all below 2%, and there was no significant difference in the values of PCE/(PCE + NCE) among the negative control and forsythin treatment groups. Combined with the results of the Ames test, CA test, and MN test, it can be inferred that forsythin may not have genotoxicity.

In addition, the safety pharmacology evaluation of forsythin was further investigated. The pharmacologic evaluation of preclinical safety mainly contains three systems: respiratory system, cardiovascular system, and central nervous system [41]. Cardiovascular system experiments are mainly performed in large animals such as dogs or monkeys. Monitoring the cardiovascular and respiratory systems of conscious animals by the telemetry system not only facilitates the comprehensive analysis of the pharmacological effects of drugs but also is in accordance with the principle of animal experiments, which contributes to accelerate the process of drug development. Johnson et al. [42] used the telemetry method to study the cardiovascular safety pharmacology of resveratrol and found that resveratrol administration had no effect on body temperature, heart rate, blood pressure, or ECG parameters. Our research showed that MP and QTcF were shorten or prolonged, with increased and decreased heart rate after treated with forsythin. However, the dogs in the negative control group also had similar phenomena, the occurrence time was transitory, and there were no dose-dependent and time-dependent manners. In regard to the change of body temperature, the change of body temperature in daytime was within 1°C, as well as the average percentage of body temperature change was less than 3%, so there was no significant difference in the body temperature index among groups. Considering the normal physiological rhythm changes in animals, the differences in respiratory rate and tidal volume were not due to the effects of forsythin. Taken together, it can be inferred that these alterations may be related to the changes in the physiological rhythm and sitting posture of animals and not associated with the forsythin treatment.

## 5. Conclusions

In conclusion, the results of the Ames test, CA assay, and in vivo MN assay for forsythin were all negative, which indicated that the clinical application of forsythin may have low probability of genetic toxicity. Additionally, no significant toxicological effects of forsythin on respiratory and cardiovascular systems in conscious beagle dogs were observed. Our findings reveal that forsythin is safe and provide an important support to better understand the biosafety of forsythin for further development and applications.

## Figures and Tables

**Table 1:** Results of the bacterial reverse mutation test (Ames test) for forsythin.

S9	Chemical	Dose (*μ*g/plate)	His^+^ revertant colonies/plate				
			TA97	TA98	TA100	TA102	TA1535
−	DMSO (1%)^a^	0	137.4 ± 30.8	16.4 ± 2.5	75.2 ± 43.5	263.8 ± 127.4	12.8 ± 0.5
	Dexon^b^	100	1609.0 ± 273.9^*∗∗*^	1122.2 ± 593.0^*∗∗*^	1429.6 ± 101.3^*∗∗*^	2253.1 ± 431.8^*∗∗*^	
	Sodium azide^b^	15					2170.2 ± 332.2^*∗∗*^
	Forsythin	2500	125.2 ± 54.2	21.0 ± 5.6	65.7 ± 51.8	333.8 ± 112.4	10.5 ± 4.4
		1250	154.0 ± 33.0	17.5 ± 4.5	53.1 ± 38.2	285.8 ± 52.8	11.8 ± 2.1
		625	128.9 ± 52.4	13.3 ± 4.7	61.4 ± 48.3	237.7 ± 118.8	11.6 ± 3.2
		312	144.8 ± 51.1	18.4 ± 9.3	61.3 ± 44.3	216.6 ± 85.3	10.2 ± 1.6
		156	135.2 ± 16.9	18.7 ± 6.0	56.3 ± 44.5	200.5 ± 42.0	11.7 ± 1.4

+	DMSO (1%)^a^	0	157.1 ± 54.7	15.7 ± 4.2	77.9 ± 57.4	318.6 ± 86.6	16.7 ± 5.0
	2-Aminofluorine^b^	30	1027.5 ± 296.2^*∗∗*^	2936.6 ± 686.4^*∗∗*^	768.7 ± 88.3^*∗∗*^		
	1, 8-Dioxyanthraquinone^b^	50				934.6 ± 69.6^*∗∗*^	
	Cyclophosphamide^b^	200					459.8 ± 178.9^*∗∗*^
	Forsythin	2500	160.4 ± 28.0	17.4 ± 6.0	79.8 ± 71.5	343.1 ± 172.3	11.6 ± 3.8
		1250	162.0 ± 39.6	21.8 ± 2.0	68.8 ± 54.0	344.9 ± 75.4	13.2 ± 0.9
		625	160.5 ± 38.3	17.5 ± 6.5	77.1 ± 72.9	274.1 ± 101.7	10.6 ± 1.9
		312	153.1 ± 42.1	19.3 ± 3.8	59.1 ± 64.0	307.9 ± 154.7	10.6 ± 4.0
		156	170.3 ± 41.3	17.4 ± 3.1	68.4 ± 56.2	278.8 ± 66.5	14.2 ± 3.4

Data are expressed as means ± standard deviation (SD). ^*∗*^*p* < 0.05, compared with the negative control group; ^*∗∗*^*p* < 0.01, compared with the negative control group. ^a^Negative control. ^b^Positive control.

**Table 2:** Results of the chromosomal aberrations test for forsythin.

Group	Dosage (*µ*g/ml)	Number of cells scored	No. of cells with aberrations		
			−S9		+S9
			24 h	48 h	6–18 h
DMSO^a^	—	200	4.0	2.5	2.0
Mitomycin C^b^	0.25	200	14.5^*∗∗*^	18.5^*∗∗*^	
Cyclophosphamide^b^	20	200			19.0^*∗∗*^
Forsythin	125	200	5.0	2.5	5.0
	250	200	4.5	1.5	5.0
	500	200	5.0	5.0	4.5

^a^Negative control. ^b^Positive control. ^*∗*^*p* < 0.05, compared with the negative control group. ^*∗∗*^*p* < 0.01, compared with the negative control group.

**Table 3:** Results of the in vivo bone marrow micronucleus test for forsythin.

Group	Dosage (mg/kg)	Number of mice	MNPCE^c^	PCE/(PCE + NCE)^d^
0.5% CMC-Na^a^	—	10	0.6 ± 0.4	44.3 ± 10.7
Cyclophosphamide^b^	80	10	13.0 ± 4.1^*∗∗*^	43.2 ± 7.6
Forsythin	4500	10	1.0 ± 0.6	55.4 ± 8.9^*∗*^
	9000	10	1.2 ± 0.8	42.6 ± 10.1
	18000	10	1.6 ± 0.8^*∗∗*^	45.1 ± 3.9

Data expressed as means ± SD. ^*∗*^*p* < 0.05, compared with the negative control group. ^*∗∗*^*p* < 0.01, compared with the negative control group. ^a^Negative control. ^b^Positive control. ^c^Micronucleated polychromatic erythrocytes (MNPCEs) calculated from 2000 polychromatic erythrocytes (%). ^d^The ratio (%) of polychromatic erythrocytes (PCE) and all erythrocytes (PCE + NEC). NEC, normochromatic erythrocytes.

## Data Availability

The datasets used and/or analyzed to support the findings of this study are available from the corresponding author upon request.

## Abbreviations

GLP: Good Laboratory Practice
OECD: Organization for Economic Cooperation and Development
CFDA: China Food and Drug Administration
ICH: International Conference on Harmonization of Technical Requirements for Registration of Pharmaceuticals for Human Use
AAALAC: Association for Assessment and Accreditation of Laboratory Animal Care
NIBP: Noninvasive blood pressure
PCE: Polychromatic erythrocyte
NCE: Normochromatic erythrocyte
MNPCEs: Micronucleated polychromatic erythrocytes
DMSO: Dimethylsulfoxide.
